# Impact of blood pressure control during pregnancy on long-term maternal cardiovascular health – secondary analysis of a follow-up study 15 years post preeclampsia (PAVA study)

**DOI:** 10.1371/journal.pone.0344378

**Published:** 2026-07-15

**Authors:** Charlotte Lößner, Anne Loheit, Anna Multhaup, Laura Bäz, Thomas Lehmann, Yvonne Heimann, Ekkehard Schleußner, Marcus Franz, Tanja Groten

**Affiliations:** 1 Department of Obstetrics, University Hospital Jena, Friedrich Schiller University Jena, Jena, Germany; 2 Center for Early Pregnancy and Reproductive Health (CEPRE), University Hospital, Friedrich Schiller University, Jena, Germany; 3 Department of Internal Medicine I, University Hospital Jena, Friedrich Schiller University Jena, Jena, Germany; 4 Institute of Medical Statistics, Computer Sciences and Documentation, University Hospital Jena, Friedrich Schiller University Jena, Jena, Germany; 5 Department of Obstetrics and Gynecology, Faculty of Medicine and University Hospital Cologne, University of Cologne, Cologne, Germany; Scuola Superiore Sant’Anna, ITALY

## Abstract

**Background:**

Within the PAVA study, we investigated placenta-associated vascular aging in women 15 years after pregnancies complicated by preeclampsia and/or fetal growth restriction. During these pregnancies, blood pressure values >140/90 mmHg were considered to require treatment. Following evidence demonstrating improved pregnancy outcomes with tighter blood pressure control, updated German and international guidelines (International Society for the Study of Hypertension in Pregnancy, ISSHP) now recommend target values below 135/85 mmHg. To evaluate whether blood pressure levels during pregnancy are associated with long-term maternal cardiovascular health, we analyzed the impact of blood pressure control during pregnancy on cardiovascular risk profiles obtained in the PAVA follow-up study.

**Methods:**

Between August 2019 and December 2022, 53 women were examined an average of 15 years after experiencing preeclampsia and/or fetal growth restriction. At the study visit, baseline data were collected, and a comprehensive clinical and functional assessment was performed. This analysis compared 35 women who were hypertensive at follow-up (blood pressure ≥140/90 mmHg and/or antihypertensive medication) with 18 normotensive participants (blood pressure <140/90 mmHg and no antihypertensive medication). Data on blood pressure levels during pregnancy were collected retrospectively from medical records.

**Results:**

Blood pressure data during pregnancy were available for the second trimester in 15 women who later developed hypertension and 4 who remained normotensive, and for the third trimester in 19 and 14 participants, respectively. Data from the first trimester were not available. In group comparisons, systolic blood pressure in the second trimester and diastolic blood pressure in the third trimester were significantly higher in women who were hypertensive at follow-up (155 vs. 140 mmHg, p = 0.027; 96 vs. 87 mmHg, p = 0.026). In adjusted analyses, no statistically significant associations were observed; however, effect estimates suggested a potential association between blood pressure control during pregnancy and lower odds of later arterial hypertension (OR 0.22, 95% CI 0.03–1.53) and concentric remodeling (OR 0.37, 95% CI 0.03–4.29).

**Conclusions:**

These findings suggest that blood pressure levels during pregnancy may be associated with long-term maternal cardiovascular health. However, given the limited sample size and exploratory nature of the analysis, these results should be interpreted with caution and require confirmation in larger prospective studies.

## Introduction

Elevated blood pressure before, during and after pregnancy affects short-term maternal and fetal outcomes as well as long-term maternal health. Elevated blood pressure during pregnancy has been shown to be associated with an increased risk of pregnancy complications, such as preeclampsia (PE) [1]. Strict blood pressure control during pregnancy in women with chronic or gestational hypertension has been shown to improve pregnancy outcome and to prevent severe maternal hypertension [2]. Women with hypertensive pregnancy diseases have an increased long-term risk of cardiovascular disease such as hypertension, cerebrovascular stroke, coronary heart disease and even heart failure [3]. It has been shown, that after adjustment for confounding factors, the risk of chronic hypertension 1–5 years after pregnancy was 4–10 times higher in women with hypertensive disorders during pregnancy than in women with normotensive pregnancies [4]. The effect of blood pressure within the recommended targets during pregnancy on long-term cardiovascular health of affected women is so far understudied. We therefore analyzed the specific effects of blood pressure control during pregnancy on the cardiovascular risk profile 15 years after pregnancies complicated by placenta associated pregnancy diseases like PE and/or FGR, using the data retrieved during our PAVA-study.

## Methods

### Design and study population

**Fig 1:**
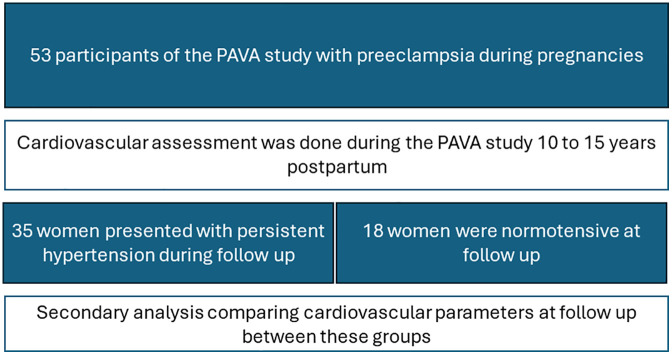
Cohort composition.

This study constitutes a subgroup analysis of the PAVA study, a prospective, cross-sectional, single-center trial conducted by our group and published in 2024 [5]. In the original PAVA cohort, 53 women with a history of PE and/or FGR and 51 women with previously uncomplicated pregnancies were assessed on average 15 years postpartum. In order to investigate the effect of blood pressure control during pregnancy affected by PE and/or FGR, we further analysed the retrieved long term follow up data of the subgroup of 53 women with a history of PE and/or FGR. Within this cohort we compared clinical characteristics and date of cardiovascular function analysis in women presenting with hypertension at the follow-up study visit and the cohort of normotensive women. (Fig 1) Hypertension was defined as presenting with systolic blood pressure values ≥ 140 mmHg and/or a diastolic blood pressure ≥ 90 mmHg and/or currently taking antihypertensive medication, whereas normotension was defined as having a systolic blood pressure below 140 mmHg and a diastolic blood pressure below 90 mmHg in the absence of antihypertensive medication. Written informed consent was obtained for each enrolled patient during the PAVA study. Data were processed in accordance with the European data safety regulations. Clinical data from the index pregnancies were retrospectively collected from hospital database and patient records where available. To ensure pseudonymized analysis of data, each patient record was given a unique patient identification number when it was entered into the study database. Ethical approval was obtained from the Ethics Committee of the Friedrich Schiller University, Jena, Germany (no. 2019–1498_BO). The PAVA trial is registered with the US National Library of Medicine Clinical- Trials.gov (No: NCT04484766).

### Clinical characteristics

Clinical characteristics and results of transthoracic echocardiography and VICORDER measurement (Skidmore Medical Ltd, Bristol, United Kingdom), were retrieved as described in detail in the original article [5]. Based on available echocardiological data, we calculated left ventricular mass (LVM), left ventricular mass index (LVMI) and relative wall thickness (RWT) analogous to the algorithm published by Hashem et al.[6] and subsequently categorized results in physiological cardiac values (RWT ≤ 0.42, LVMI < 95 g/m^2^), concentric cardiac remodelling (RWT > 0.42, LVMI < 95 g/m^2^), concentric cardiac hypertrophy (RWT > 0.42, LVMI ≥ 95 g/m^2^) or eccentric cardiac hypertrophy (RWT ≤ 0.42, LVMI ≥ 95 g/m^2^).

To improve comparability, blood pressure values retrieved from obstetric records were documented as the minimum and maximum as well as the mean value for each trimester of pregnancy. During pregnancy, blood pressure was measured by medical staff according to standard criteria.

### Statistical analysis

Categorical variables were compared between groups and are reported as absolute and relative frequencies. Continuous variables were analyzed using the non-parametric Mann–Whitney U test, as appropriate due to the distributional characteristics of the data and the relatively small sample size. Accordingly, medians as well as 25th and 75th percentiles are reported for continuous variables. All tests were two-sided.

Group characteristics such as maternal BMI prior to pregnancy, gestational age at delivery, and blood pressure within target range during pregnancy (≤135/85 mmHg) were used as independent variables in binary logistic regression analysis. Binary logistic regression with the Wald backward elimination method was applied. Results were considered statistically significant at p < 0.05. Statistical analyses were performed using IBM SPSS Statistics version 28.0 (IBM Corp., Armonk, NY, USA).

## Results

We examined 53 women with a history of pregnancies complicated by PE and/or FGR. At the time of the study visit, 35 women (66%) were hypertensive, while 18 women (34%) were normotensive. Both systolic and diastolic mean oscillometric blood pressure were significantly higher in the group of hypertensive women than in the normotensive group (systolic 146 mmHg vs. 123 mmHg, p < 0.001; diastolic 94.5 mmHg vs. 80 mmHg, p < 0.001). A summary of selected group characteristics at the PAVA follow-up study visit is presented in Table 1.

**Table 1:** Group characteristics at PAVA-study follow-up visit of 53 women after pregnancies complicated by PE and/or FGR*.

	Women with hypertension^1^ (N = 35)	Women without hypertension^1^ (N = 18)	p**
**Group characteristics at study visit**			
Years since index pregnancy	14 (12/16)	14 (11.5/15.5)	0.477
Age^(years)^	44 (41/47)	41 (39.5/48)	0.400
BMI^(kg/m2)^	29.41 (24.7/35.49)	23.1 (21.12/27.31)	0.003
BP systolic^(mmHg)^	146 (135/158)	123 (117.75/129)	**<0.001**
BP diastolic^(mmHg)^	94.5 (88/98.5)	80 (73.75/83.75)	**<0.001**
Current chronic diseases			
Diabetes mellitus	2 (5.7%)	–	0.320
Thyroid Diseases	9 (25.7%)	3 (17.6%)	0.521
Lipid Metabolism Diseases	1 (2.9%)	–	0.486
Mental Diseases	2 (5.7%)	–	0.320
Other Diseases	11 (31.4%)	5 (29.4%)	0.884
On Medication			
Antihypertensive medication	22 (62.9%)	–	**<0.001**
Other medication^2^	21 (60%)	7 (41.2%)	0.206
Consumption behaviour			
Smoking	5 (14.3%)	5 (29.4%)	**0.004**
Alcohol consumption	23 (65.7%)	15 (88.2%)	0.089
Drug consumption	1 (5.9%)	1 (5.9%)	0.151
Level of Education^4^			
Low	5 (14.3%)	–	0.127
Middle	20 (57.1%)	5 (33.3%)	0.127
High	10 (28.6%)	10 (66.7%)	**0.013**
IPAQ^5^			
Low physical activity	11 (37.9%)	–	**0.004**
Moderate physical activity	8 (27.6%)	6 (35.3%)	0.588
High physical activity	10 (34.5%)	11 (64.7%)	**0.049**
BIA			
BIA fm^(%)^	38.2 (32.25/40.3), N = 33	30 (24.2/38.2), N = 16	**0.013**
BIA bcm^(%)^	33.1 (31.7/36.25), N = 33	38.2 (32.45/41.73), N = 16	**0.046**

*Data are n (%) or median (25^th^-75^th^ percentile). Number of subjects (N) is given if deviating from indicated group size. Significant results by Mann-Whitney-U-Test (*p* < 0.05) are highlighted in bold. **comparing women after PE/FGR with hypertension at study visit vs. women after PE/FGR without hypertension at study visit; ^1^current intake of antihypertensive medication and/or blood pressure ≥ 140/90 mmHg at study visit; ^2^other medication including hormonal contraceptives, thyroid hormones, antidiabetics, blood thinners, diuretics, psychotropic drugs, antiepileptics, glucocorticoid sprays, antihistamines, NSAIDs, PPI, dietary supplements and herbal preparations; ^3^all blood pressure values recorded during pregnancy ≤ 135/85 mmHg; ^4^level of education low, middle school education; level of education middle, high school education; level of education high, general qualification for university entrance; ^5^IPAQ high physical activity, vigorous intensity activity on at least 3 days achieving a minimum total physical activity of at least 1500 MET minutes a week or 7 or more days of any combination of walking, moderate intensity or vigorous intensity activities achieving a minimum total physical activity of at least 3000 MET minutes a week; IPAQ moderate physical activity, 3 or more days of vigorous intensity activity and/or walking of at least 30 minutes per day or 5 or more days of moderate intensity activity and/or walking of at least 30 minutes per day 5 or more days of any combination of walking, moderate intensity or vigorous intensity activities achieving a minimum total physical activity of at least 600 MET minutes a week; IPAQ low physical activity, if not meeting any of criteria for either moderate of high levels of physical activity; PE, preeclampsia; FGR, fetal growth restriction; BP, blood pressure; BMI, body mass index; IPAQ, International Physical Activity Questionnaire; BIA, bioelectrical impendance analysis; BIA fm, bioelectrical impendance analysis fat mass; BIA bcm, bioelectrical impendance analysis body cell mass

Blood pressure measurements retrieved during the second trimester of pregnancy were available for 15 women who subsequently developed hypertension and for 4 women who remained normotensive. Data from the third trimester were available for 19 and 14 women, respectively. In group comparisons, women who later developed hypertension exhibited higher mean blood pressure values during pregnancy. This difference reached statistical significance for systolic blood pressure in the second trimester (155 mmHg vs. 140 mmHg, p = 0.027) and for diastolic blood pressure in the third trimester (96 mmHg vs. 87 mmHg, p = 0.026). Overall, a significantly smaller proportion of women in the group that later developed hypertension had gestational blood pressure values within the target range of ≤135/85 mmHg (6.7% vs. 35.3%, p = 0.013). A summary of blood pressure values and additional characteristics before and during the index pregnancy is provided in Table 2.

**Table 2:** Pregnancy characteristics of 53 women from the PAVA-study cohort after pregnancies complicated by PE and/or FGR*.

	Women with hypertension^1^ at study visit (N = 35)	Women without hypertension^1^ at study visit (N = 18)	p**
**Group characteristics prior to index pregnancy**			
Chronic diseases prior to index pregnancy			
Arterial hypertension Other Diseases	9 (30%), N = 30	0 (0%)	**0.011**
	14 (46.7%), N = 30	6 (33.3%)	0.369
Medication intake prior to index pregnancy			
Antihypertensive medication Other medication^2^	3 (10%), N = 30	0 (0%)	0.170
	7 (20%)	2 (11.1%)	0.419
**Group characteristics during index pregnancy**			
Maternal Age at delivery^(years)^	31 (27/34)	31 (27/34)	0.914
Maternal BMI prior to pregnancy ^(kg/m2)^	23.83 (21.69/29.93), N = 28	21.54 (20.25/23.62), N = 16	**0.025**
Gestational age at delivery^(weeks of pregnancy)^	33.5 (28/36), N = 34	35 (30/37.5)	0.199
Birth Weight percentile child^(%)^	8 (2/16.5), N = 33	4 (2/10.5)	0.485
Medication intake during pregnancy			
Antihypertensive medication Other medication^2^	10 (28.6%)	2 (11.8%)	0.181
	16 (45.7%)	5 (29.4%)	0.266
BP during pregnancy			
BP systolic 1^st^ trimester^(mmHg)^ BP diastolic 1^st^ trimester^(mmHg)^ BP systolic 2^nd^ trimester^(mmHg)^ BP diastolic 2^nd^ trimester^(mmHg)^ BP systolic 3^rd^ trimester^(mmHg)^ BP diastolic 3^rd^ trimester^(mmHg)^	129.5 (129.5/129.5), N = 1	112 (112/112), N = 1	1.00
	77 (77/77), N = 1	62 (62/62), N = 1	1.00
	155 (145/167), N = 15	140.5 (126.88/145.88), N = 4	**0.027**
	93.5 (90/97.5), N = 15	90.75 (77.88/98.75), N = 4	0.665
	142.5 (135/160), N = 19	136.25 (114.88/152.38), N = 14	0.271
	96.5 (90/100), N = 19	87.5 (74.88/94.75), N = 14	**0.026**
Blood pressure in target range during pregnancy^3^	2 (6.7%), N = 30	6 (35.3%), N = 17	**0.013**

*Data are n (%) or median (25^th^-75^th^ percentile). Number of subjects (N) is given if deviating from indicated group size. Significant results by Mann-Whitney-U-Test (*p* < 0.05) are highlighted in bold. **comparing women after PE/FGR with hypertension at study visit vs. women after PE/FGR without hypertension at study visit; ^1^current intake of antihypertensive medication and/or blood pressure ≥ 140/90 mmHg at study visit; ^2^other medication including hormonal contraceptives, thyroid hormones, antidiabetics, blood thinners, diuretics, psychotropic drugs, antiepileptics, glucocorticoid sprays, antihistamines, NSAIDs, PPI, dietary supplements and herbal preparations; ^3^all blood pressure values recorded during pregnancy ≤ 135/85 mmHg; PE, preeclampsia; FGR, fetal growth restriction; BP, blood pressure; BMI, body mass index

Table 3 summarizes parameters of cardiovascular function. Physiological cardiac values were present in 51.6% of hypertensive and 66.7% of normotensive women (p = 0.340). Echocardiographic assessment revealed no significant differences in left heart structure, except for a reduced left ventricular ejection fraction in hypertensive women (2CH: 63.5% vs. 67.5%, p = 0.02; 4CH: 62% vs. 66.5%, p = 0.036) and a thicker interventricular septum (11 mm vs. 10 mm, p = 0.05). The E/med E ratio was significantly elevated in hypertensive subjects (9.8 vs. 7.9, p = 0.006). Regarding right heart morphology, hypertensive women exhibited a larger right atrial area (13 cm² vs. 11 cm², p = 0.036), with no further differences observed. Rates of concentric remodelling (38.7% vs. 20%, p = 0.209), concentric hypertrophy (6.5% vs. 6.7%, p = 0.978), and eccentric hypertrophy (3.2% vs. 6.7%, p = 0.596) did not differ significantly between groups, although a non-significant trend toward higher prevalence of concentric remodelling was noted among hypertensive women.

**Table 3:** Results of cardiovascular function analysis at PAVA-study follow-up visit of 53 women after pregnancies complicated by PE and/or FGR *.

	Women with hypertension^1^ (N = 35)	Women without hypertension^1^ (N = 18)	p**
**Transthoracic echocardiography**			
HR^(bpm)^	68.5 (63.5/78.25), N = 30	66 (63/73), N = 13	0.610
CO^(l/min)^	3.54 (3.13/4.59), N = 30	3.74 (2.58/4.5), N = 14	0.465
SV^(ml)^	53 (39.5/62.25), N = 30	57.5 (45.74/66.25), N = 14	0.753
LVEDD M^(mm)^	45 (42/48), N = 31	42.5 (39.75/48.25), N = 14	0.389
LVEDD 4CH(^mm)^	44 (42/48), N = 31	43 (41/46), N = 14	0.505
LVESD M^(mm)^	27 (24/29), N = 31	27.5 (23.75/29.25), N = 14	0.685
LVESD 4CH^(mm)^	29 (27/32), N = 31	27 (25.75/28.25), N = 14	0.052
LVPWD M^(mm)^	9 (8/10), N = 31	8.5 (8/10.25), N = 14	0.511
LA area^(cm2)^	18 (14/18), N = 31	15.5 (12.75/18.25), N = 14	0.165
LA volume^(ml)^	46 (32/50), N = 31	35.5 (27/45.5), N = 14	0.064
RVEDD^(mm)^	35 (31/37), N = 31	33.5 (29/35), N = 14	0.140
RVESD^(mm)^	23 (20/25), N = 31	21 (20/26), N = 14	0.863
RA area^(cm2)^	13 (11/15), N = 31	11 (9.75/13.25), N = 14	**0.036**
lVSD^(mm)^	11 (10/11), N = 31	10 (8.75/10.25), N = 14	**0.050**
lVSD 4CH^(mm)^	10 (9/12), N = 31	9 (8.75/10), N = 14	0.117
LVEF 2CH^(%)^	63.5 (60/66), N = 30	67.5 (61.25/69.5), N = 12	**0.020**
LVEF 4CH^(%)^	62 (58/66), N = 30	66.5 (63/68), N = 14	**0.036**
TAPSE^(mm)^	23 (20/25), N = 27	22 (20/26), N = 11	0.849
sPAP^(mmHg)^	19 (13.5/23), N = 22	17 (16/22), N = 9	0.949
RVOT^(mm)^	28 (25/30), N = 30	26 (23/30.5), N = 13	0.232
PH^(mm)^	20 (19/22.75), N = 20	18.5 (17.75/22.25), N = 10	0.286
IVC^(mm^)	13 (11/14), N = 31	14 (11/18), N = 13	0.508
PVR^(WU)^	1.05 (0.78/1.68), N = 23	1.14 (1.17/1.58), N = 8	0.362
TRV^(m/s)^	2.01 (1.83/2.41), N = 22	2.1 (1.99/2.34), N = 9	0.814
TVI RVOT^(cm)^	17 (14.75/22), N = 26	17 (13/19), N = 11	0.256
E/A	1.45 (1.08/1.7), N = 30	1.6 (1.46/1.78), N = 14	0.106
DCT^(ms)^	231.5 (195.75/251.75), N = 30	226 (187.75/269.75), N = 14	0.614
E/ med E	9.8 (8.53/11.68), N = 28	7.9 (6.67/8.5), N = 12	**0.006**
E/ lat E	6.65 (5.4/8.4), N = 24	6.4 (4.65/7.27), N = 9	0.349
Diastolic Dysfunction	8 (28.6%)	1 (9.1%)	0.363
LVM^(g)^	147 (126.7/163), N = 31	113.58 (93.46/164.32), N = 15	0.122
LVMI^(g/m2)^	76.83 (68.55/84), N = 31	66.64 (56.15/84.17), N = 15	0.186
LVMI >95	3 (9.7%), N = 31	2 (13.3%), N = 15	0.712
RWT	0.41 (0.36/0.47), N = 31	0.4 (0.35/0.45), N = 15	0.631
RWT > 0,42	14 (45.2%), N = 31	4 (26.7%), N = 15	0.233
Physiological cardiac values	16 (51.6%), N = 31	10 (66.7%), N = 15	0.340
Concentric remodeling	12 (38.7%), N = 31	3 (20%), N = 15	0.209
Concentric hypertrophy	2 (6.5%), N = 31	1 (6.7%), N = 15	0.978
Eccentric hypertrophy	1 (3.2%), N = 31	1 (6.7%), N = 15	0.596
**VICORDER**			
PP ^(mmHg)^	69 (64/80)	64 (57/67.5)	**0.005**
PWV^(m/s)^	6 (4/8)	6 (4/8.5)	0.757
Aix	26 (21/30)	21 (14.5/27.5)	**0.014**
AoPP^(mmHg)^	66 (63/78)	61 (53.5/63)	**<0.001**
AoBP sys^(mmHg)^	144 (135/155)	123 (117.5/126.5)	**<0.001**
AoBP dia^(mmHg)^	76 (68/82)	62 (58/68)	**<0.001**
MAP^(mmHg)^	106 (98/115)	91 (85/93.5)	**<0.001**
SV^(ml)^	113 (101/137)	110 (95/116.5)	0.160
CO^(l/min)^	8 (7/9)	7 (6/7)	**0.003**
CI^(l/min/m2)^	4 (4/5)	4 (3/4)	0.073
SEVR^(%)^	142 (131/161)	151 (147/161.5)	0.163
TPR^(PRU)^	0.85 (0.72/0.94), N = 33	0.83 (0.75/0.97)	0.552
FMS^(%)^	13.5 (8.75/21), N = 34	13 (8.25/22.75)	0.654

*Data are n (%) or median (25^th^-75^th^ percentile). Number of subjects (N) is given if deviating from indicated group size. Significant results by Mann-Whitney-U-Test (*p* < 0.05) are highlighted in bold; **comparing women after PE/FGR with hypertension at study visit vs. women after PE/FGR without hypertension at study visit; PE, preeclampsia; FGR, fetal growth restriction; ^1^current intake of antihypertensive medication and/or blood pressure ≥ 140/90 mmHg at study visit; HR, heart rate; CO, cardiac output; SV, stroke volume; M, M-mode; 4CH, 4-chamber view; 2CH, 2-chamber view; LVEDD, left ventricular end-diastolic diameter; LVESD, left ventricular end-systolic diameter; LVPWD, left ventricular rear wall diameter; LA area, left atrial area; LA volume, left atrial volume; RVEDD, right ventricular end-diastolic diameter; RVESD, right ventricular end-systolic diameter; RA area, right atrial area; lVSD, interventricular septum diameter; LVEF, left ventricular ejection fraction; TAPSE, tricuspid annular plane systolic excursion; sPAP, systolic pulmonary arterial pressure; RVOT, right ventricular outflow tract; PH, pulmonary trunk diameter; IVC, inferior vena cava diameter; PVR, pulmonary vascular resistance; TRV, tricuspid regurgitant velocity; TVI RVOT, time-velocity integral of right ventricular outflow tract; E/A, E/A ratio; DCT, deceleration time; E/ med e´, E-wave velocity/ medial e´-velocity- ratio; E/ lat e´, E-wave velocity/ lateral e´-velocity-ratio; Diastolic Disfunction, existence of diastolic dysfunction; LVM, left ventricular mass (0.8 (1.04 [LVEDD+LVPWD+IVSD]^3^– [LVEDD]^3^) + 0.6 g); LVMI, left ventricular mass index (LVM/ body surface area; reference range female < 95 g/m^2^); RWT, relative wall thickness (2* LVPWD/LVEDD; reference range male and female ≤ 0.42); Physiological cardiac values (RWT ≤ 0.42, LVMI < 95 g/m^2^); Concentric remodelling (RWT > 0.42, LVMI < 95 g/m^2^); Concentric hypertrophy (RWT > 0.42, LVMI ≥ 95 g/m^2^); Eccentric hypertrophy (RWT ≤ 0.42, LVMI ≥ 95 g/m^2^); PP, pulse pressure; PWV, pulse wave velocity; Aix, augmentation index; AoPP, aortic pulse pressure; AoBP sys, aortic blood pressure systolic; AoBP dia, aortic blood pressure diastolic; MAP, mean arterial pressure; CI, cardiac index; SEVR, subendocardial viability ratio; TPR, total peripheral resistance; FMS, flow mediated slowing.

VICORDER analysis demonstrated significantly higher aortic systolic (144 vs. 123 mmHg, p < 0.001) and diastolic blood pressure (76 vs. 62 mmHg, p < 0.001), increased pulse pressure (peripheral: 69 vs. 64 mmHg, p = 0.005; aortic: 66 vs. 61 mmHg, p < 0.001), and elevated mean arterial pressure (106 vs. 91 mmHg, p < 0.001) in hypertensive women. At comparable pulse wave velocity, the augmentation index was significantly higher in the hypertensive group (26 vs. 21, p = 0.014). Cardiac output was also increased, averaging 8 l/min compared with 7 l/min in normotensive women (p = 0.003).

Table 4 presents adjusted odds ratios (ORs) and corresponding confidence intervals (CIs) for selected long-term maternal cardiovascular outcomes, including arterial hypertension, diastolic dysfunction, abnormal relative wall thickness (RWT), and concentric remodelling. The models were adjusted for maternal pre-pregnancy body mass index (BMI), gestational age at delivery, and blood pressure control during pregnancy.

**Table 4:** Adjusted odds ratios (ORs) for long-term maternal cardiovascular complications.

	Arterial hypertension ^a^		Diastolic Dysfunction ^b^		Abnormal RWT ^c^		Concentric remodelling ^d^	
	OR (CI)	p	OR (CI)	p	OR (CI)	p	OR (CI)	p
Maternal BMI prior to pregnancy ^(kg/m2)^	1.11 (0.95-1.3)	0.183	1.06 (0.87-1.30)	0.553	1.05 (0.9-1.22)	0.536	1.01 (0.86-1.18)	0.927
Gestational age at delivery^(weeks of pregnancy)^	0.99 (0.83-1.19)	0.915	0.88 (0.70-1.11)	0.287	0.97 (0.8-1.17)	0.726	0.99 (0.81-1.2)	0.875
Blood pressure in target range during pregnancy ^(all blood pressure values recorded during pregnancy ≤ 135/85 mmHg)^	0.22 (0.03-1.53)	0.125	1.20 (0.08-18.32)	0.894	0.93 (0.12-7.31)	0.945	0.37 (0.03-4.29)	0.423

Adjustments were made for maternal BMI prior to pregnancy, gestational age at delivery and blood pressure in target range during pregnancy. Number of included cases in modell: a n = 41; b n = 10; c n = 13; d n = 13. BMI, body mass index; RWT, relative wall thickness.

## Discussion

This study examines the impact of blood pressure control during pregnancy on cardiovascular risk profiles 15 years following pregnancies complicated by PE and/or FGR. While our findings suggest a potential association between lower blood pressure values during pregnancy and more favorable long-term cardiovascular outcomes, these results should be interpreted with caution given the exploratory nature of the study and the limited sample size. Previous studies have demonstrated that adequate blood pressure control improves short-term maternal and fetal outcomes [2,7], and our data may indicate that this effect could extend to long-term maternal health.

Women with lower blood pressure values in the second and third trimesters tended to show a lower risk of arterial hypertension 15 years after a complicated pregnancy. The data obtained during the study visit — indicating a higher educational level, greater habitual physical activity, and more favorable BIA parameters in the group of normotensive women — suggest the presence of an additive lifestyle effect following pregnancy (Table 1). However, these baseline differences between groups, including body mass index, smoking behavior, education level, and physical activity, represent important cardiovascular risk factors and may have influenced the observed associations. Although adjustments were performed, residual confounding cannot be excluded.

The relatively small sample size and limited number of events reduce statistical power, which is reflected in the wide confidence intervals observed in binary logistic regression analyses. Accordingly, none of the investigated variables emerged as statistically significant predictors of long-term cardiovascular complications in adjusted models. Although maintenance of blood pressure within the target range (≤ 135/85 mmHg) was associated with a trend toward reduced odds of postpartum arterial hypertension and concentric remodelling (Table 4), these findings cannot be considered conclusive and should be regarded as hypothesis-generating.

In addition, the assessment of blood pressure control during pregnancy was limited by incomplete data. Blood pressure measurements from the first trimester were not available, and documentation in later trimesters was variable. The definition of “blood pressure within the target range” required all recorded values to remain below a predefined threshold, representing a relatively strict criterion. In the context of incomplete or infrequent measurements, this approach may have led to misclassification and may have influenced the observed associations.

Concentric remodeling refers to a structural adaptation of the left ventricle characterized by increased wall thickness without a concomitant increase in overall left ventricular mass. This pattern typically arises as a compensatory response to chronic pressure overload, such as that induced by hypertension, and is linked to an elevated risk of cardiovascular morbidity and mortality, even in the absence of overt left ventricular hypertrophy. Our results suggest an association between lower blood pressure values during pregnancy and a reduced risk of later concentric remodeling, which is consistent with findings from similar studies [3,8]. However, these studies primarily focused on hypertensive pregnancy complications as a whole rather than on blood pressure control during pregnancy itself.

Also, VICORDER analysis revealed group differences. The group of hypertensive women exhibited increased systolic aortic blood pressure in combination with increased peripheral and aortic pulse pressure, exceeding clinically accepted reference limits (Table 3). An elevated pulse pressure indicates increased vascular stiffness and is associated with cardiovascular risk. A meta-analysis showed that, for every 10 mmHg increase in pulse pressure, the relative risk of cardiovascular mortality increases by 13%, while the relative risk of all-cause mortality increases by 9% [9]. In this context, our findings suggest a potentially unfavorable vascular profile in women who remain hypertensive 15 years after pregnancy; however, causal inferences cannot be drawn.

### Strengths and limitations

This study has several limitations. Its retrospective design and the relatively small sample size limit statistical power and generalizability of the findings. In addition, incomplete blood pressure data, particularly from the first trimester, restrict the assessment of blood pressure control throughout pregnancy and may have introduced misclassification. The number of statistical comparisons performed in relation to the sample size also raises the possibility of type I error, and no formal adjustment for multiple testing was applied. Therefore, the results, particularly those approaching statistical significance, should be interpreted with caution.

Despite these limitations, the study has important strengths. It is based on a well-characterized cohort of women who underwent comprehensive cardiovascular assessment approximately 15 years after preeclampsia. The parallel use of transthoracic echocardiography and VICORDER measurements enabled an in-depth evaluation of both cardiovascular structure and function as well as vascular properties. This comprehensive phenotyping provides a robust dataset for the analysis of long-term cardiovascular sequelae following preeclampsia.

## Conclusions

Collectively, our findings suggest that blood pressure control during pregnancy may be associated with long-term maternal cardiovascular health. They highlight the importance of close blood pressure monitoring and management during pregnancy to reduce perinatal complications and potentially influence long-term risk. However, given the exploratory nature of the study and its methodological limitations, these findings should be interpreted cautiously. Future research should focus on larger, prospective studies investigating optimal strategies and timing of blood pressure management during pregnancy, with the aim of improving both short- and long-term maternal outcomes.
