# Supplementary material for: Impact of blood pressure control during pregnancy on long-term maternal cardiovascular health – secondary analysis of a follow-up study 15 years post preeclampsia (PAVA study)
Source: PLoS One. 2026 Jul 15;21(7):e0344378. doi: 10.1371/journal.pone.0344378 (PMC13372152; doi:10.1371/journal.pone.0344378)
Supplement: Attachment — Submitted filename: renamed_ee882.docx [file pone.0344378.s002.docx]

# **Response to Reviewer**

**We sincerely thank the reviewer for the detailed and constructive comments. We have carefully revised the Discussion and related sections to address all concerns. The changes are outlined below with reference to the revised manuscript paragraphs.**

**___________________________________________________________________________**

**Reviewer #1:** This manuscript presents a secondary analysis of the PAVA study investigating whether blood pressure control during pregnancies complicated by preeclampsia and/or fetal growth restriction influences long-term maternal cardiovascular health approximately 15 years postpartum. The authors analyzed a subgroup of 53 women and compared those who were hypertensive at follow-up with those who were normotensive. Blood pressure values during pregnancy were retrospectively retrieved from medical records. Cardiovascular outcomes were assessed using comprehensive clinical evaluation, transthoracic echocardiography, and VICORDER measurements. Statistical analyses included non-parametric group comparisons and multivariable logistic regression models adjusted for pre-pregnancy BMI, gestational age at delivery, and blood pressure control during pregnancy.

Overall, the article addresses a clinically relevant and timely question. The idea of linking the degree of blood pressure control during pregnancy with long-term cardiovascular outcomes is important and relevant in light of evolving treatment targets.

The methodology appears appropriate, cardiovascular phenotyping at follow-up is comprehensive, the statistical analyses are generally adequate, and the results are clearly described.

**Nevertheless, several issues should be addressed:**

1. Limited sample size and statistical power.

The analyses are based on a relatively small cohort, with very small subgroup sizes for trimester-specific blood pressure data. Some regression models include few events, resulting in wide confidence intervals. While the authors acknowledge the lack of statistical significance, the Discussion occasionally suggests a protective effect despite non-significant findings. The interpretation should be more cautious and clearly framed as exploratory.

**Response:**
We thank the reviewer for this important comment. The Discussion has been revised to more clearly reflect the exploratory nature of the study and to avoid overinterpretation.

- In **Discussion, lines 183-190,** we replaced stronger wording (e.g., “support the hypothesis”) with more cautious phrasing (“suggest a potential association” and “should be interpreted with caution”).
- In **Discussion, lines 201-202,** we explicitly state that the small sample size and limited number of events reduce statistical power and result in wide confidence intervals.
- We further clarify that none of the investigated variables reached statistical significance and that observed associations should be considered **hypothesis-generating rather than conclusive, lines 204-208**.

2. Potential confounding and baseline group differences

Women who were hypertensive at follow-up differed significantly in BMI, smoking behavior, education level, and physical activity (Table 1). These variables are strongly associated with cardiovascular risk and may confound the observed associations. Adjustment was limited, and residual confounding cannot be excluded. A more detailed discussion of these differences and their potential impact on the findings would strengthen the manuscript.

**Response:**
We agree and have expanded the Discussion accordingly.

- In **Discussion, lines 191-200**, we now explicitly describe the observed differences in BMI, smoking behavior, education level, and physical activity between groups.
- We emphasize that these are established cardiovascular risk factors and may act as confounders.
- We also added that **residual confounding cannot be excluded**, despite adjustment **(lines 199-200).**

3. Definition and completeness of blood pressure control during pregnancy

Blood pressure data during pregnancy were incomplete (e.g., no first-trimester data, missing values in later trimesters). The definition of “blood pressure in target range” (all recorded values ≤135/85 mmHg) appears strict and may lead to misclassification, especially in women with sparse documentation. The authors should discuss how missing data and variable documentation frequency might have influenced the categorization and results.

**Response:**
We appreciate this important point and have added a dedicated paragraph addressing these limitations.

- In **Discussion, lines 209-2015,** we now discuss:
  - Missing first-trimester blood pressure data
  - Variable documentation frequency in later trimesters
  - The strict definition of “blood pressure within the target range”
- We explicitly state that this approach may have led to **misclassification** and influenced the results **(lines 213-215).**

4. Separation of Results and Discussion

In the Discussion, the authors repeatedly restate specific numerical results (e.g., odds ratios and p-values, as well as explicit references to Table 1 and Table 4). In my view, detailed reporting of numerical findings belongs primarily in the Results section, whereas the Discussion should focus more on interpretation, contextualization, and implications. Reiterating exact ORs and p-values in the Discussion can blur the structural distinction between sections and may give additional weight to statistically non-significant findings.

I would recommend streamlining the Discussion by summarizing the direction and magnitude of effects without repeating detailed statistics, and by placing greater emphasis on interpretation rather than result reporting.

**Response:**
We agree and have revised the Discussion to improve structural clarity.

- Detailed numerical results (odds ratios, p-values) have been removed from the Discussion.
- Direct emphasis on tables has been reduced.
- The revised Discussion now focuses on **interpretation and contextualization rather than result reporting** throughout all paragraphs.

In summary, this is a well-conceived and clinically relevant exploratory study with appropriate methodology and clearly presented results. Addressing the points above—particularly by ensuring cautious interpretation and clearer structural separation between Results and Discussion—would further strengthen the manuscript.

**___________________________________________________________________________**

**Reviewer #2:** The current study goal is to evaluate whether blood pressure values during pregnancy impact on maternal long-term cardiovascular health and to analyze the effect of blood pressure control during pregnancy on the cardiovascular risk profile obtained during the PAVA study. Obviously, there is no sample size calculation as this is a retrospective evaluation.

1. However, the study does have the limit of being subject to poor design conditions and perhaps having an insufficient sample to properly address the objectives.

**Response:**
We thank the reviewer for this important comment.

- We have strengthened the discussion of study limitations accordingly.
- In **Strengths and Limitations, lines 237-244,** we now explicitly state that the retrospective design and relatively small sample size limit statistical power and generalizability.
- Additionally, in the **Discussion (lines 183-190, lines 201-208)** and **Conclusions (lines 253-261)**, we emphasize the exploratory nature of the study and that the findings should be interpreted with caution.

2. Also in the abstract what is the meaning of the phrase,’ Furthermore, although due to the small sample size not reaching significance, adjusted analysis did …….. etc’? Reaching what significance? There was no calculated sample size to reach any effect size goals. What effects, differences , etc. were the investigators striving for, if any?

**Response:**
We agree and have revised the Abstract to improve clarity and avoid misleading interpretation.

- The wording referring to “not reaching significance” has been removed/rephrased. We now clearly state that **no statistically significant associations were observed in adjusted analyses**, without implying a predefined significance threshold or target effect size **(lines 50-51).**

3. Using non-parametric analyses implies that the sample size was anticipated to be small.

**Response:**
We thank the reviewer for this observation.

- Non-parametric tests were chosen due to the distributional characteristics of the data and the relatively small sample size.
- We have clarified this rationale in the **Statistical Analysis section**, stating that non-parametric methods were applied where appropriate due to sample size and non-normal distribution of variables **(lines 125-127)**.

4. Also in the Statistical analysis section and later in the analysis results what ‘binary regression’ was used? Presumably logistic regression? Please be specific as to what analysis is being used when presenting the results?

**Response:**
We thank the reviewer for pointing this out.

- The term “binary regression” has been replaced with **“binary logistic regression”** throughout the manuscript, including the **Statistical Analysis section** and the **Results section**, to ensure clarity and precision **(line 132, line 205)**.

5. Using the term, ’hypothesis’ in the Conclusion, lines 58-60 in the Abstract may be inappropriate. Perhaps the term , ‘goal’, or ‘aim’ may be more appropriate.

**Response:**
We thank the reviewer for this helpful comment.

- We agree that the term “hypothesis” may imply a level of confirmatory inference that is not appropriate for the exploratory nature of this study.
- We have therefore revised the Abstract accordingly. The term “hypothesis” has been removed and replaced with more appropriate terminology reflecting the study’s exploratory aim and objective.
- In addition, the concluding statements have been rephrased to avoid causal or confirmatory language and now appropriately reflect the observational design of the study **(lines 55-58)**.

6. There are many comparisons being made in Tables 1 to 3 for such a small sample. This could be a multiple comparisons issue and there is no mention made of p-value adjustments. This , well as the small sample, should be listed as a limitation in the ‘Discussion’ section and the reader notified that the significant results should be interpreted with caution.

**Response:**
We thank the reviewer for highlighting this important issue.

- We have added a corresponding limitation in **Strengths and Limitations, (lines 240-247)**, stating that the number of statistical comparisons relative to the sample size raises the possibility of **type I error**, and that no formal adjustment for multiple testing was performed. We further emphasize that results, particularly those approaching statistical significance, should be interpreted with caution.

The paper requires a good edit to address the above or related issues.

**___________________________________________________________________________**

**We again thank the reviewer for the insightful comments, which have significantly improved the clarity and rigor of our manuscript. We believe that the revisions have addressed all concerns and strengthened the overall quality of the study.**
