# Supplementary material for: Impact of blood pressure control during pregnancy on long-term maternal cardiovascular health – secondary analysis of a follow-up study 15 years post preeclampsia (PAVA study)
Source: PLoS One. 2026 Jul 15;21(7):e0344378. doi: 10.1371/journal.pone.0344378 (PMC13372152; doi:10.1371/journal.pone.0344378)
Supplement: Peer review history

# Peer review: Decision Letter 0

**DOI:** 10.1371/journal.pone.0344378.r001

**Authors:** Vincenzo Lionetti

11 Apr 2026

PONE-D-26-04324

Impact of blood pressure control during pregnancy on long-term maternal cardiovascular health – secondary analysis of a follow-up study 15 years post preeclampsia (PAVA study)

PLOS One

Dear Dr. Groten,

Thank you for submitting your manuscript to PLOS ONE. After careful consideration, we feel that it has merit but does not fully meet PLOS ONE’s publication criteria as it currently stands. Therefore, we invite you to submit a revised version of the manuscript that addresses the points raised during the review process.

Please submit your revised manuscript by May 26 2026 11:59PM. If you will need more time than this to complete your revisions, please reply to this message or contact the journal office at plosone@plos.org. When you're ready to submit your revision, log on to https://www.editorialmanager.com/pone/ and select the 'Submissions Needing Revision' folder to locate your manuscript file.

Please include the following items when submitting your revised manuscript:

- A letter that responds to each point raised by the academic editor and reviewer(s). You should upload this letter as a separate file labeled 'Response to Reviewers'.
- A marked-up copy of your manuscript that highlights changes made to the original version. You should upload this as a separate file labeled 'Revised Manuscript with Track Changes'.
- An unmarked version of your revised paper without tracked changes. You should upload this as a separate file labeled 'Manuscript'.

If you would like to make changes to your financial disclosure, please include your updated statement in your cover letter. Guidelines for resubmitting your figure files are available below the reviewer comments at the end of this letter.

If applicable, we recommend that you deposit your laboratory protocols in protocols.io to enhance the reproducibility of your results. Protocols.io assigns your protocol its own identifier (DOI) so that it can be cited independently in the future. For instructions see: https://journals.plos.org/plosone/s/submission-guidelines#loc-laboratory-protocols. Additionally, PLOS ONE offers an option for publishing peer-reviewed Lab Protocol articles, which describe protocols hosted on protocols.io. Read more information on sharing protocols at https://plos.org/protocols?utm_medium=editorial-email&utm_source=authorletters&utm_campaign=protocols.

As the corresponding author, your ORCID iD is verified in the submission system and will appear in the published article. PLOS supports the use of ORCID, and we encourage all coauthors to register for an ORCID iD and use it as well. Please encourage your coauthors to verify their ORCID iD within the submission system before final acceptance, as unverified ORCID iDs will not appear in the published article. *Only*  the individual author can complete the verification step; PLOS staff *cannot*  verify ORCID iDs on behalf of authors.

We look forward to receiving your revised manuscript.

Kind regards,

Vincenzo Lionetti, M.D., PhD

Academic Editor

PLOS One

Journal Requirements:

When submitting your revision, we need you to address these additional requirements.

1.Please ensure that your manuscript meets PLOS ONE's style requirements, including those for file naming. The PLOS ONE style templates can be found at

https://journals.plos.org/plosone/s/file?id=wjVg/PLOSOne_formatting_sample_main_body.pdf and

https://journals.plos.org/plosone/s/file?id=ba62/PLOSOne_formatting_sample_title_authors_affiliations.pdf

2. For studies involving third-party data, we encourage authors to share any data specific to their analyses that they can legally distribute. PLOS recognizes, however, that authors may be using third-party data they do not have the rights to share. When third-party data cannot be publicly shared, authors must provide all information necessary for interested researchers to apply to gain access to the data. (https://journals.plos.org/plosone/s/data-availability#loc-acceptable-data-access-restrictions)

3. We notice that your supplementary figures are uploaded with the file type 'Figure'. Please amend the file type to 'Supporting Information'. Please ensure that each Supporting Information file has a legend listed in the manuscript after the references list.

4. If the reviewer comments include a recommendation to cite specific previously published works, please review and evaluate these publications to determine whether they are relevant and should be cited. There is no requirement to cite these works unless the editor has indicated otherwise.

Additional Editor Comments:

all issues raised by expert reviewers are required.

[Note: HTML markup is below. Please do not edit.]

Reviewer's Responses to Questions

-->Comments to the Author

1. Is the manuscript technically sound, and do the data support the conclusions?

The manuscript must describe a technically sound piece of scientific research with data that supports the conclusions. Experiments must have been conducted rigorously, with appropriate controls, replication, and sample sizes. The conclusions must be drawn appropriately based on the data presented.

Reviewer #1: Yes

Reviewer #2: Partly

**********

2. Has the statistical analysis been performed appropriately and rigorously?

Reviewer #1: Yes

Reviewer #2: No

**********

3. Have the authors made all data underlying the findings in their manuscript fully available?

The PLOS Data policy requires authors to make all data underlying the findings described in their manuscript fully available without restriction, with rare exception (please refer to the Data Availability Statement in the manuscript PDF file). The data should be provided as part of the manuscript or its supporting information, or deposited to a public repository. For example, in addition to summary statistics, the data points behind means, medians and variance measures should be available. If there are restrictions on publicly sharing data—e.g. participant privacy or use of data from a third party—those must be specified.

Reviewer #1: Yes

Reviewer #2: Yes

**********

4. Is the manuscript presented in an intelligible fashion and written in standard English?

PLOS ONE does not copyedit accepted manuscripts, so the language in submitted articles must be clear, correct, and unambiguous. Any typographical or grammatical errors should be corrected at revision, so please note any specific errors here.

Reviewer #1: Yes

Reviewer #2: Yes

**********

5. Review Comments to the Author

Please use the space provided to explain your answers to the questions above. You may also include additional comments for the author, including concerns about dual publication, research ethics, or publication ethics. (Please upload your review as an attachment if it exceeds 20,000 characters)

Reviewer #1: This manuscript presents a secondary analysis of the PAVA study investigating whether blood pressure control during pregnancies complicated by preeclampsia and/or fetal growth restriction influences long-term maternal cardiovascular health approximately 15 years postpartum. The authors analyzed a subgroup of 53 women and compared those who were hypertensive at follow-up with those who were normotensive. Blood pressure values during pregnancy were retrospectively retrieved from medical records. Cardiovascular outcomes were assessed using comprehensive clinical evaluation, transthoracic echocardiography, and VICORDER measurements. Statistical analyses included non-parametric group comparisons and multivariable logistic regression models adjusted for pre-pregnancy BMI, gestational age at delivery, and blood pressure control during pregnancy.

Overall, the article addresses a clinically relevant and timely question. The idea of linking the degree of blood pressure control during pregnancy with long-term cardiovascular outcomes is important and relevant in light of evolving treatment targets. The methodology appears appropriate, cardiovascular phenotyping at follow-up is comprehensive, the statistical analyses are generally adequate, and the results are clearly described.

Nevertheless, several issues should be addressed:

Limited sample size and statistical power

The analyses are based on a relatively small cohort, with very small subgroup sizes for trimester-specific blood pressure data. Some regression models include few events, resulting in wide confidence intervals. While the authors acknowledge the lack of statistical significance, the Discussion occasionally suggests a protective effect despite non-significant findings. The interpretation should be more cautious and clearly framed as exploratory.

Potential confounding and baseline group differences

Women who were hypertensive at follow-up differed significantly in BMI, smoking behavior, education level, and physical activity (Table 1). These variables are strongly associated with cardiovascular risk and may confound the observed associations. Adjustment was limited, and residual confounding cannot be excluded. A more detailed discussion of these differences and their potential impact on the findings would strengthen the manuscript.

Definition and completeness of blood pressure control during pregnancy

Blood pressure data during pregnancy were incomplete (e.g., no first-trimester data, missing values in later trimesters). The definition of “blood pressure in target range” (all recorded values ≤135/85 mmHg) appears strict and may lead to misclassification, especially in women with sparse documentation. The authors should discuss how missing data and variable documentation frequency might have influenced the categorization and results.

Separation of Results and Discussion

In the Discussion, the authors repeatedly restate specific numerical results (e.g., odds ratios and p-values, as well as explicit references to Table 1 and Table 4). In my view, detailed reporting of numerical findings belongs primarily in the Results section, whereas the Discussion should focus more on interpretation, contextualization, and implications. Reiterating exact ORs and p-values in the Discussion can blur the structural distinction between sections and may give additional weight to statistically non-significant findings. I would recommend streamlining the Discussion by summarizing the direction and magnitude of effects without repeating detailed statistics, and by placing greater emphasis on interpretation rather than result reporting.

In summary, this is a well-conceived and clinically relevant exploratory study with appropriate methodology and clearly presented results. Addressing the points above—particularly by ensuring cautious interpretation and clearer structural separation between Results and Discussion—would further strengthen the manuscript.

Reviewer #2: The current study goal is to evaluate whether blood pressure values during pregnancy impact on maternal long-term cardiovascular health and to analyze the effect of blood pressure control during pregnancy on the cardiovascular risk profile obtained during the PAVA study. Obviously there is no sample size calculation as this is a retrospective evaluation.

1. However, the study does have the limit of being subject to poor design conditions and perhaps having an insufficient sample to properly address the objectives.

2. Also in the abstract what is the meaning of the phrase,’ Furthermore, although due to the small sample size not reaching significance, adjusted analysis did …….. etc’? Reaching what significance? There was no calculated sample size to reach any effect size goals. What effects, differences , etc. were the investigators striving for, if any?

3. Using non parametric analyses implies that the sample size was anticipated to be small.

4. Also in the Statistical analysis section and later in the analysis results what ‘binary regression’ was used? Presumably logistic regression? Please be specific as to what analysis is being used when presenting the results?

5. Using the term, ’hypothesis’ in the Conclusion, lines 58-60 in the Abstract may be inappropriate. Perhaps the term , ‘goal’, or ‘aim’ may be more appropriate.

6. There are many comparisons being made in Tables 1 to 3 for such a small sample. This could be a multiple comparisons issue and there is no mention made of p-value adjustments. This , well as the small sample, should be listed as a limitation in the ‘Discussion’ section and the reader notified that the significant results should be interpreted with caution.

The paper requires a good edit to address the above or related issues.

**********

6. PLOS authors have the option to publish the peer review history of their article (what does this mean?). If published, this will include your full peer review and any attached files.

If you choose “no”, your identity will remain anonymous but your review may still be made public.

Do you want your identity to be public for this peer review? For information about this choice, including consent withdrawal, please see our Privacy Policy.

Reviewer #1: No

Reviewer #2: No

**********

[NOTE: If reviewer comments were submitted as an attachment file, they will be attached to this email and accessible via the submission site. Please log into your account, locate the manuscript record, and check for the action link "View Attachments". If this link does not appear, there are no attachment files.]

To ensure your figures meet our technical requirements, please review our figure guidelines: https://journals.plos.org/plosone/s/figures

You may also use PLOS’s free figure tool, NAAS, to help you prepare publication quality figures: https://journals.plos.org/plosone/s/figures#loc-tools-for-figure-preparation.

NAAS will assess whether your figures meet our technical requirements by comparing each figure against our figure specifications.

# Author comment: Author response to Decision Letter 1

**DOI:** 10.1371/journal.pone.0344378.r002

27 May 2026

Response to Reviewer

We sincerely thank the reviewer for the detailed and constructive comments. We have carefully revised the Discussion and related sections to address all concerns. The changes are outlined below with reference to the revised manuscript paragraphs.

___________________________________________________________________________

Reviewer #1: This manuscript presents a secondary analysis of the PAVA study investigating whether blood pressure control during pregnancies complicated by preeclampsia and/or fetal growth restriction influences long-term maternal cardiovascular health approximately 15 years postpartum. The authors analyzed a subgroup of 53 women and compared those who were hypertensive at follow-up with those who were normotensive. Blood pressure values during pregnancy were retrospectively retrieved from medical records. Cardiovascular outcomes were assessed using comprehensive clinical evaluation, transthoracic echocardiography, and VICORDER measurements. Statistical analyses included non-parametric group comparisons and multivariable logistic regression models adjusted for pre-pregnancy BMI, gestational age at delivery, and blood pressure control during pregnancy.

Overall, the article addresses a clinically relevant and timely question. The idea of linking the degree of blood pressure control during pregnancy with long-term cardiovascular outcomes is important and relevant in light of evolving treatment targets.

The methodology appears appropriate, cardiovascular phenotyping at follow-up is comprehensive, the statistical analyses are generally adequate, and the results are clearly described.

Nevertheless, several issues should be addressed:

1. Limited sample size and statistical power.

The analyses are based on a relatively small cohort, with very small subgroup sizes for trimester-specific blood pressure data. Some regression models include few events, resulting in wide confidence intervals. While the authors acknowledge the lack of statistical significance, the Discussion occasionally suggests a protective effect despite non-significant findings. The interpretation should be more cautious and clearly framed as exploratory.

Response:

We thank the reviewer for this important comment. The Discussion has been revised to more clearly reflect the exploratory nature of the study and to avoid overinterpretation.

• In Discussion, lines 183-190, we replaced stronger wording (e.g., “support the hypothesis”) with more cautious phrasing (“suggest a potential association” and “should be interpreted with caution”).

• In Discussion, lines 201-202, we explicitly state that the small sample size and limited number of events reduce statistical power and result in wide confidence intervals.

• We further clarify that none of the investigated variables reached statistical significance and that observed associations should be considered hypothesis-generating rather than conclusive, lines 204-208.

2. Potential confounding and baseline group differences

Women who were hypertensive at follow-up differed significantly in BMI, smoking behavior, education level, and physical activity (Table 1). These variables are strongly associated with cardiovascular risk and may confound the observed associations. Adjustment was limited, and residual confounding cannot be excluded. A more detailed discussion of these differences and their potential impact on the findings would strengthen the manuscript.

Response:

We agree and have expanded the Discussion accordingly.

• In Discussion, lines 191-200, we now explicitly describe the observed differences in BMI, smoking behavior, education level, and physical activity between groups.

• We emphasize that these are established cardiovascular risk factors and may act as confounders.

• We also added that residual confounding cannot be excluded, despite adjustment (lines 199-200).

3. Definition and completeness of blood pressure control during pregnancy

Blood pressure data during pregnancy were incomplete (e.g., no first-trimester data, missing values in later trimesters). The definition of “blood pressure in target range” (all recorded values ≤135/85 mmHg) appears strict and may lead to misclassification, especially in women with sparse documentation. The authors should discuss how missing data and variable documentation frequency might have influenced the categorization and results.

Response:

We appreciate this important point and have added a dedicated paragraph addressing these limitations.

• In Discussion, lines 209-2015, we now discuss:

o Missing first-trimester blood pressure data

o Variable documentation frequency in later trimesters

o The strict definition of “blood pressure within the target range”

• We explicitly state that this approach may have led to misclassification and influenced the results (lines 213-215).

4. Separation of Results and Discussion

In the Discussion, the authors repeatedly restate specific numerical results (e.g., odds ratios and p-values, as well as explicit references to Table 1 and Table 4). In my view, detailed reporting of numerical findings belongs primarily in the Results section, whereas the Discussion should focus more on interpretation, contextualization, and implications. Reiterating exact ORs and p-values in the Discussion can blur the structural distinction between sections and may give additional weight to statistically non-significant findings.

I would recommend streamlining the Discussion by summarizing the direction and magnitude of effects without repeating detailed statistics, and by placing greater emphasis on interpretation rather than result reporting.

Response:

We agree and have revised the Discussion to improve structural clarity.

• Detailed numerical results (odds ratios, p-values) have been removed from the Discussion.

• Direct emphasis on tables has been reduced.

• The revised Discussion now focuses on interpretation and contextualization rather than result reporting throughout all paragraphs.

In summary, this is a well-conceived and clinically relevant exploratory study with appropriate methodology and clearly presented results. Addressing the points above—particularly by ensuring cautious interpretation and clearer structural separation between Results and Discussion—would further strengthen the manuscript.

___________________________________________________________________________

Reviewer #2: The current study goal is to evaluate whether blood pressure values during pregnancy impact on maternal long-term cardiovascular health and to analyze the effect of blood pressure control during pregnancy on the cardiovascular risk profile obtained during the PAVA study. Obviously, there is no sample size calculation as this is a retrospective evaluation.

1. However, the study does have the limit of being subject to poor design conditions and perhaps having an insufficient sample to properly address the objectives.

Response:

We thank the reviewer for this important comment.

• We have strengthened the discussion of study limitations accordingly.

• In Strengths and Limitations, lines 237-244, we now explicitly state that the retrospective design and relatively small sample size limit statistical power and generalizability.

• Additionally, in the Discussion (lines 183-190, lines 201-208) and Conclusions (lines 253-261), we emphasize the exploratory nature of the study and that the findings should be interpreted with caution.

2. Also in the abstract what is the meaning of the phrase,’ Furthermore, although due to the small sample size not reaching significance, adjusted analysis did …….. etc’? Reaching what significance? There was no calculated sample size to reach any effect size goals. What effects, differences , etc. were the investigators striving for, if any?

Response:

We agree and have revised the Abstract to improve clarity and avoid misleading interpretation.

• The wording referring to “not reaching significance” has been removed/rephrased. We now clearly state that no statistically significant associations were observed in adjusted analyses, without implying a predefined significance threshold or target effect size (lines 50-51).

3. Using non-parametric analyses implies that the sample size was anticipated to be small.

Response:

We thank the reviewer for this observation.

• Non-parametric tests were chosen due to the distributional characteristics of the data and the relatively small sample size.

• We have clarified this rationale in the Statistical Analysis section, stating that non-parametric methods were applied where appropriate due to sample size and non-normal distribution of variables (lines 125-127).

4. Also in the Statistical analysis section and later in the analysis results what ‘binary regression’ was used? Presumably logistic regression? Please be specific as to what analysis is being used when presenting the results?

Response:

We thank the reviewer for pointing this out.

• The term “binary regression” has been replaced with “binary logistic regression” throughout the manuscript, including the Statistical Analysis section and the Results section, to ensure clarity and precision (line 132, line 205).

5. Using the term, ’hypothesis’ in the Conclusion, lines 58-60 in the Abstract may be inappropriate. Perhaps the term , ‘goal’, or ‘aim’ may be more appropriate.

Response:

We thank the reviewer for this helpful comment.

• We agree that the term “hypothesis” may imply a level of confirmatory inference that is not appropriate for the exploratory nature of this study.

• We have therefore revised the Abstract accordingly. The term “hypothesis” has been removed and replaced with more appropriate terminology reflecting the study’s exploratory aim and objective.

• In addition, the concluding statements have been rephrased to avoid causal or confirmatory language and now appropriately reflect the observational design of the study (lines 55-58).

6. There are many comparisons being made in Tables 1 to 3 for such a small sample. This could be a multiple comparisons issue and there is no mention made of p-value adjustments. This , well as the small sample, should be listed as a limitation in the ‘Discussion’ section and the reader notified that the significant results should be interpreted with caution.

Response:

We thank the reviewer for highlighting this important issue.

• We have added a corresponding limitation in Strengths and Limitations, (lines 240-247), stating that the number of statistical comparisons relative to the sample size raises the possibility of type I error, and that no formal adjustment for multiple testing was performed. We further emphasize that results, particularly those approaching statistical significance, should be interpreted with caution.

The paper requires a good edit to address the above or related issues.

___________________________________________________________________________

We again thank the reviewer for the insightful comments, which have significantly improved the clarity and rigor of our manuscript. We believe that the revisions have addressed all concerns and strengthened the overall quality of the study.

Attachment Submitted filename: renamed_ee882.docx

# Peer review: Decision Letter 1

**DOI:** 10.1371/journal.pone.0344378.r003

**Authors:** Vincenzo Lionetti

1 Jun 2026

Impact of blood pressure control during pregnancy on long-term maternal cardiovascular health – secondary analysis of a follow-up study 15 years post preeclampsia (PAVA study)

PONE-D-26-04324R1

Dear Dr. Groten,

We’re pleased to inform you that your manuscript has been judged scientifically suitable for publication and will be formally accepted for publication once it meets all outstanding technical requirements.

Within one week, you’ll receive an e-mail detailing the required amendments. When these have been addressed, you’ll receive a formal acceptance letter and your manuscript will be scheduled for publication.

An invoice will be generated when your article is formally accepted. Please note, if your institution has a publishing partnership with PLOS and your article meets the relevant criteria, all or part of your publication costs will be covered. Please make sure your user information is up-to-date by logging into Editorial Manager at Editorial Manager® and clicking the ‘Update My Information' link at the top of the page. For questions related to billing, please contact billing support.

If your institution or institutions have a press office, please notify them about your upcoming paper to help maximize its impact. If they’ll be preparing press materials, please inform our press team as soon as possible -- no later than 48 hours after receiving the formal acceptance. Your manuscript will remain under strict press embargo until 2 pm Eastern Time on the date of publication. For more information, please contact onepress@plos.org.

Kind regards,

Vincenzo Lionetti, M.D., PhD

Academic Editor

PLOS One

Additional Editor Comments (optional):

Reviewers' comments:

Reviewer's Responses to Questions

**Comments to the Author**

1. If the authors have adequately addressed your comments raised in a previous round of review and you feel that this manuscript is now acceptable for publication, you may indicate that here to bypass the “Comments to the Author” section, enter your conflict of interest statement in the “Confidential to Editor” section, and submit your "Accept" recommendation.

Reviewer #1: All comments have been addressed

Reviewer #2: All comments have been addressed

**********

2. Is the manuscript technically sound, and do the data support the conclusions?

The manuscript must describe a technically sound piece of scientific research with data that supports the conclusions. Experiments must have been conducted rigorously, with appropriate controls, replication, and sample sizes. The conclusions must be drawn appropriately based on the data presented.

Reviewer #1: Yes

Reviewer #2: (No Response)

**********

3. Has the statistical analysis been performed appropriately and rigorously?

Reviewer #1: Yes

Reviewer #2: (No Response)

**********

4. Have the authors made all data underlying the findings in their manuscript fully available?

The PLOS Data policy requires authors to make all data underlying the findings described in their manuscript fully available without restriction, with rare exception (please refer to the Data Availability Statement in the manuscript PDF file). The data should be provided as part of the manuscript or its supporting information, or deposited to a public repository. For example, in addition to summary statistics, the data points behind means, medians and variance measures should be available. If there are restrictions on publicly sharing data—e.g. participant privacy or use of data from a third party—those must be specified.

Reviewer #1: Yes

Reviewer #2: (No Response)

**********

5. Is the manuscript presented in an intelligible fashion and written in standard English?

PLOS ONE does not copyedit accepted manuscripts, so the language in submitted articles must be clear, correct, and unambiguous. Any typographical or grammatical errors should be corrected at revision, so please note any specific errors here.

Reviewer #1: Yes

Reviewer #2: (No Response)

**********

6. Review Comments to the Author

Please use the space provided to explain your answers to the questions above. You may also include additional comments for the author, including concerns about dual publication, research ethics, or publication ethics. (Please upload your review as an attachment if it exceeds 20,000 characters)

Reviewer #1: (No Response)

Reviewer #2: (No Response)

**********

7. PLOS authors have the option to publish the peer review history of their article (what does this mean?). If published, this will include your full peer review and any attached files.

If you choose “no”, your identity will remain anonymous but your review may still be made public.

**Do you want your identity to be public for this peer review?**  For information about this choice, including consent withdrawal, please see our Privacy Policy.

Reviewer #1: **Yes:** Ulrich Pecks

Reviewer #2: No

**********

# Editor report: Acceptance letter

**DOI:** 10.1371/journal.pone.0344378.r004

**Authors:** Vincenzo Lionetti

PONE-D-26-04324R1

PLOS One

Dear Dr. Groten,

I'm pleased to inform you that your manuscript has been deemed suitable for publication in PLOS One. Congratulations! Your manuscript is now being handed over to our production team.

At this stage, our production department will prepare your paper for publication. This includes ensuring the following:

* All references, tables, and figures are properly cited

* All relevant supporting information is included in the manuscript submission,

* There are no issues that prevent the paper from being properly typeset

You will receive further instructions from the production team, including instructions on how to review your proof when it is ready. Please keep in mind that we are working through a large volume of accepted articles, so please give us a few days to review your paper and let you know the next and final steps.

Lastly, if your institution or institutions have a press office, please let them know about your upcoming paper now to help maximize its impact. If they'll be preparing press materials, please inform our press team within the next 48 hours. Your manuscript will remain under strict press embargo until 2 pm Eastern Time on the date of publication. For more information, please contact onepress@plos.org.

You will receive an invoice from PLOS for your publication fee after your manuscript has reached the completed accept phase. If you receive an email requesting payment before acceptance or for any other service, this may be a phishing scheme. Learn how to identify phishing emails and protect your accounts at https://explore.plos.org/phishing.

If we can help with anything else, please email us at customercare@plos.org.

Thank you for submitting your work to PLOS ONE and supporting open access.

Kind regards,

PLOS ONE Editorial Office Staff

on behalf of

Prof. Vincenzo Lionetti

Academic Editor

PLOS One
